# Operational Principles for the Dynamics of the *In Vitro* ParA-ParB System

**DOI:** 10.1371/journal.pcbi.1004651

**Published:** 2015-12-15

**Authors:** Lavisha Jindal, Eldon Emberly

**Affiliations:** Physics Department, Simon Fraser University, Burnaby, British Columbia, Canada; Max Planck Institute for Biophysical Chemistry, GERMANY

## Abstract

In many bacteria the ParA-ParB protein system is responsible for actively segregating DNA during replication. ParB proteins move by interacting with DNA bound ParA-ATP, stimulating their unbinding by catalyzing hydrolysis, that leads to rectified motion due to the creation of a wake of depleted ParA. Recent *in vitro* experiments have shown that a ParB covered magnetic bead can move with constant speed over a DNA covered substrate that is bound by ParA. It has been suggested that the formation of a gradient in ParA leads to diffusion-ratchet like motion of the ParB bead but how it forms and generates a force is still a matter of exploration. Here we develop a deterministic model for the *in vitro* ParA-ParB system and show that a ParA gradient can spontaneously form due to any amount of initial spatial noise in bound ParA. The speed of the bead is independent of this noise but depends on the ratio of the range of ParA-ParB force on the bead to that of removal of surface bound ParA by ParB. We find that at a particular ratio the speed attains a maximal value. We also consider ParA rebinding (including cooperativity) and ParA surface diffusion independently as mechanisms for ParA recovery on the surface. Depending on whether the DNA covered surface is undersaturated or saturated with ParA, we find that the bead can accelerate persistently or potentially stall. Our model highlights key requirements of the ParA-ParB driving force that are necessary for directed motion in the *in vitro* system that may provide insight into the *in vivo* dynamics of the ParA-ParB system.

## Author Summary

Segregating genetic material is essential for cell survival over multiple generations. The process underlying the required spatio-temporal organization of DNA is mediated by the ParA-ParB-parS system. Recently, experiments have shown that directed motion can be reconstituted *in vitro*. In these experiments, a magnetic bead was covered with the protein ParB and was able to move ballistically over a surface of DNA that was bound by the protein ParA. How does this active transport spontaneously emerge? In this paper we present a deterministic model for the dynamics of ParA-ParB proteins. We show how spatial noise in surface bound ParA is sufficient for the creation of a gradient in ParA that can drive motion of ParB *in vitro*. The model explains certain key aspects of the *in vitro* ParA-ParB system and leads to testable predictions.

## Introduction

A variety of mechanisms exist within bacteria to spatially localize proteins within the small confines of a bacterial cell, from reaction diffusion processes that set up waves [1] to spatial occlusion due to the highly crowded environment [2–5]. One such protein system that has been observed to display highly dynamic spatial localization within bacteria is the ParA-ParB system. These two proteins are responsible for actively transporting DNA, whether it be the replicating chromosome [6, 7] or much smaller plasmids [8], within the cell. With respect to the chromosome, it has been observed that ParB bound to sites near the origin of replication processively moves from one pole to the other via a gradient in ParA that is bound on the nucleoid surface [9]. When the system is used to segregate plasmids, ParB bound plasmids are seen to move along the ParA bound nucleoid, eventually settling into positions that are equally spaced along the cell length [10].

The exact mechanism by which the ParA-ParB system generates directed transport is not entirely resolved. Biochemical experiments have shown that ParA dimerizes in the presence of ATP and is able to to bind to DNA as ParA-ATP [11]. ParB is able to bind a specific DNA sequence known as parS and aids the autohydrolysis of ParA-ATP, causing it to unbind from the DNA [12, 13]. Recent studies have also shown that the structure of the bacterial nucleoid plays a role as well. Specifically, it has been shown that conformational changes in the nucleoid can disrupt plasmid positioning [14] and a model suggests that chromosomal elasticity could provide the required translocation force that transports partition complexes [15].

Based on the experimental evidence, several models have emerged to explain the operation of the ParA-ParB-parS system and rule out mechanisms that generate predictions which are inconsistent with observations. One model assumes that ParA-ATP binds into longitudinal filaments over the nucleoid, and that ParB initiates depolymerisation and is then carried along by a depolymerisation force [16, 17]. The filamentous structure provides directionality to the segregation of DNA. Another model considers that ParA dimers bind uniformly over the nucleoid surface and that ParB linked DNA moves via a diffusion-ratchet mechanism as it creates a wake of ParA [18]. A chemotactic force is hypothesized to exist between the two proteins that biases the otherwise free diffusion of ParB bound DNA [19]. Recent work argues that the precise nature of how ParA binds to the nucleoid surface is inconsequential for the equi-positioning of plasmids in-vivo [14]. It showed that the plasmid’s ability to move along a gradient in ParA concentration was sufficient to explain their resulting positioning. Additionally a diffusion/immobilization mechanism where freely diffusing ParB complexes are immobilized through interactions with ParA was also ruled out. Another model has ruled out freely diffusing ParB complex biased by ParA concentrations by computing that such a mechanism was insufficient to provide directionality to the motion of the partition complex [15].

Recent experimental work has managed to amazingly reconstitute the ParA-ParB system *in vitro* which provides a new context in which to explore the mechanism of how this two protein system produces active transport. Specifically, it was found that a ParB coated magnetic bead can undergo directed motion when confined to move along the plane (without rolling) of a DNA coated flow cell that was bound with ParA-ATP protein [20, 21]. Multiple beads which were identically prepared were observed to show either directed motion with differing speeds or underwent free diffusion. The beads with directed motion had diffusion constants that were around 3 times lower than that of the freely diffusing beads and had paths with persistence lengths which were many times (∼20) the radius of the micron sized bead. ParA in the vicinity of the bead was hydrolyzed and released from the surface as the bead moved and was later recovered when the bead had moved away. After an initial lag time, the bead would begin to move across the surface, creating a wake in ParA. Thus from the experimental observations, persistent motion seems to result from the creation of a ParA gradient that can provide a chemotactic force which can drive the ParB covered bead [19]. However, how does this gradient form? And how does the motion of the bead depend on the various system parameters?

**Fig 1:**
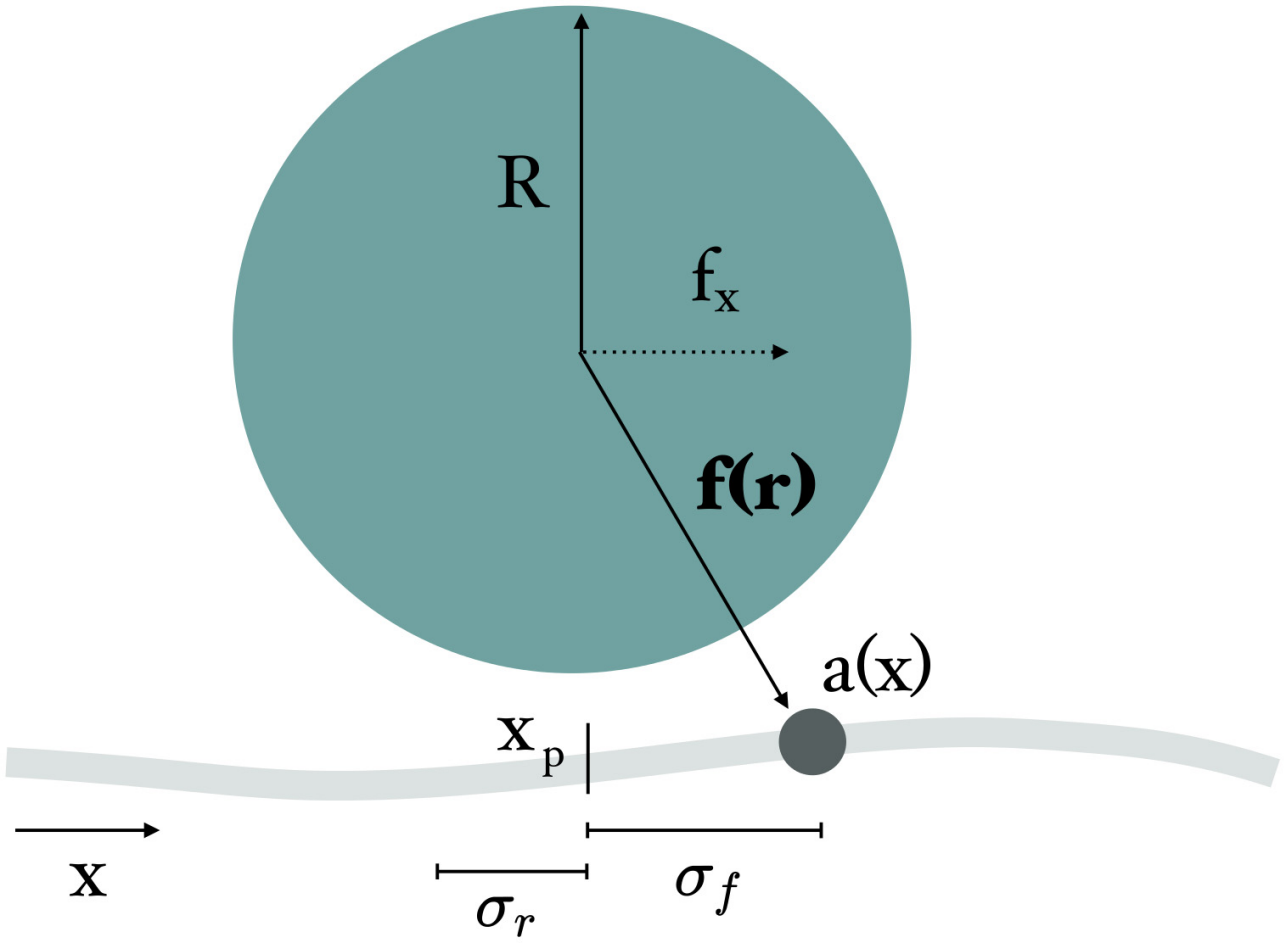
Schematic illustration of the ParA-ParB model. A ParB decorated bead of radius *R* is attracted by a central force to surface bound ParA at a location, *x*, with a concentration given by *a*(*x*, *τ*). This force decays with distance from the bead. The position of the bead is given by *x*_*p*_, and we assume that its speed is proportional to the total force acting on it.

Here we develop a completely deterministic model for the operation of the *in vitro* ParA-ParB system that complements prior modelling work on the *in vivo* system. We consider the ParB decorated bead to be an over-damped particle under the influence of attractive forces from ParA proteins on the surface (see Fig 1). As shown experimentally, beads experiencing directed motion had little diffusion [21], and so we consider a bead’s motion to be completely deterministic and proportional to this chemical force. ParA kinetics on the surface is also completely deterministic and is driven by the presence of the bead which removes ParA within its vicinity. The only noise we consider is in the initial spatial distribution of ParA and we find that this is sufficient to generate a spontaneous ParA gradient which can drive the motion of the bead. Under most conditions, we find that the bead moves with constant speed, and that this depends on the ratio of the range of the force to that of ParA removal by ParB. Interestingly, we find that the bead can attain a maximum speed which depends on this ratio.

We also consider ParA rebinding to the surface since in the *in vitro* experiment, depleted ParA regions would recover once the bead had moved away [21]. ParA surface diffusion may also serve as a possible mechanism for recovery of ParA. There are two regimes for ParA recovery on the surface: undersaturated, where there are excess binding sites on the DNA for ParA or saturated, where there is more free ParA than there are binding sites on the surface. We find that in the undersaturated regime persistent acceleration of the bead is possible. In the saturated regime, depending on the rate of rebinding and the degree of ParA cooperativity, the bead can be made to stall. From our modeling we find that distinguishing cooperative binding or ParA surface diffusion from that of non-cooperative rebinding using the current *in vitro* assay would be challenging as their effects on bead motion are all qualitatively similar. Nevertheless, the model does make predictions that could be readily testable using the *in vitro* system and suggests ways to tune the operation of the ParA-ParB system.

## Results

### Deterministic model for ParA-ParB motion

In Fig 1 we show a schematic of our minimal model for the *in vitro* ParA-ParB system. In the experiment by Vecchiarelli et al. [21], a ParB decorated bead was put into contact with a surface that was covered with strands of DNA on which ParA-ATP was bound. The observed motion of the bead was predominantly unidirectional, and so we begin by considering a 1D model for the system. We represent the surface bound ParA-ATP with a concentration, *a*(*x*, *τ*). It is initialized with a mean concentration that fluctuates uniformly from position to position with a magnitude *δa*. The bead of radius *R* is located at a position *x*_*p*_, and we assume there is a central force that acts on it due to the interactions between ParA-ATP and ParB. ParB on the bead also stimulates the removal of ParA-ATP in the vicinity of the bead. We assume that bead motion is in the overdamped regime so that the drag force balances the net force due to ParA-ParB interaction. Both the chemotactic force and the rate of removal decay with distance from the center of the bead. In the absence of surface diffusion or ParA rebinding the system dynamics for our minimal model are given by the following deterministic equations(for further details, see Methods):

$$\begin{array}{c} \frac{\partial a(x,\tau )}{\partial \tau }=-e^{-{\left(x-x_{p}\right)}^{2}/2c^{2}}a\left(x,\tau \right), \end{array}$$ (1)

$$\begin{array}{c} v=\frac{dx_{p}}{d\tau }=A_{0}\int dx\;e^{-{\left(x-x_{p}\right)}^{2}/2}\frac{x-x_{p}}{\sqrt{1+{\left(x-x_{p}\right)}^{2}}}a\left(x,\tau \right). \end{array}$$ (2)

Here the parameter *A*_0_ combines several system parameters: the initial mean ParA concentration, the amount of ParB on a bead, the ParA-ParB interaction force, and the drag on the bead (see Methods). The parameter *c* is the ratio of the lengthscale over which the bead removes nearby ParA-ATP to the lengthscale over which it experiences a force due to the surface bound ParA. We assume that both the rate of removal and force decay as Gaussian functions and *c* is the ratio of their standard deviations *σ*_*r*_ and *σ*_*f*_ respectively. Physically, *c* should be less than 1, since ParA can not be removed over distances greater than that from which it exerts an attractive force on the bead.

An estimate for these two lengthscales can be inferred from several experimental observations. First, it was found from *in vivo* measurements that active motion of plasmids require a ratio of ParB to ParA of around 5 to 1 (there are about 580 ParB bound proteins to 120 ParA in a cell [15]). We assume that the *in vitro* system requires a similar ratio to generate motion of the ParB decorated beads. The experiment conducted by Vecchiarelli et. al [21] estimates the number of ParB molecules on a bead that can interact with ParA on the surface to be 4800. They also found the number of ParA molecules present per square micron on the surface near the bead to be 400. Given the required ratio of ParA to ParB molecules, a bead with 4800 ParB molecules would need to interact with ∼ 1000 ParA molecules to generate motion. Given the ParA molecule surface density, 1000 ParA molecules would cover 1000/400 ∼ 2.5 μm^2^ of the surface leading to an effective force range, *σ*_*f*_ ∼ 0.9 μm. From the experiment, it was also observed that the radius over which the bead removed ParA from the surface (*σ*_*r*_ in our model) was on average 225 nm. Thus given these experimental observations, we estimate that *c* = *σ*_*r*_/*σ*_*f*_ ∼ 0.2−0.4 given the lower and upper bounds on the measured values. As we will show below, this value for *c* is sufficient for generating directed motion of a bead.

### Spatial noise in bound ParA is sufficient to initiate bead motion

**Fig 2:**
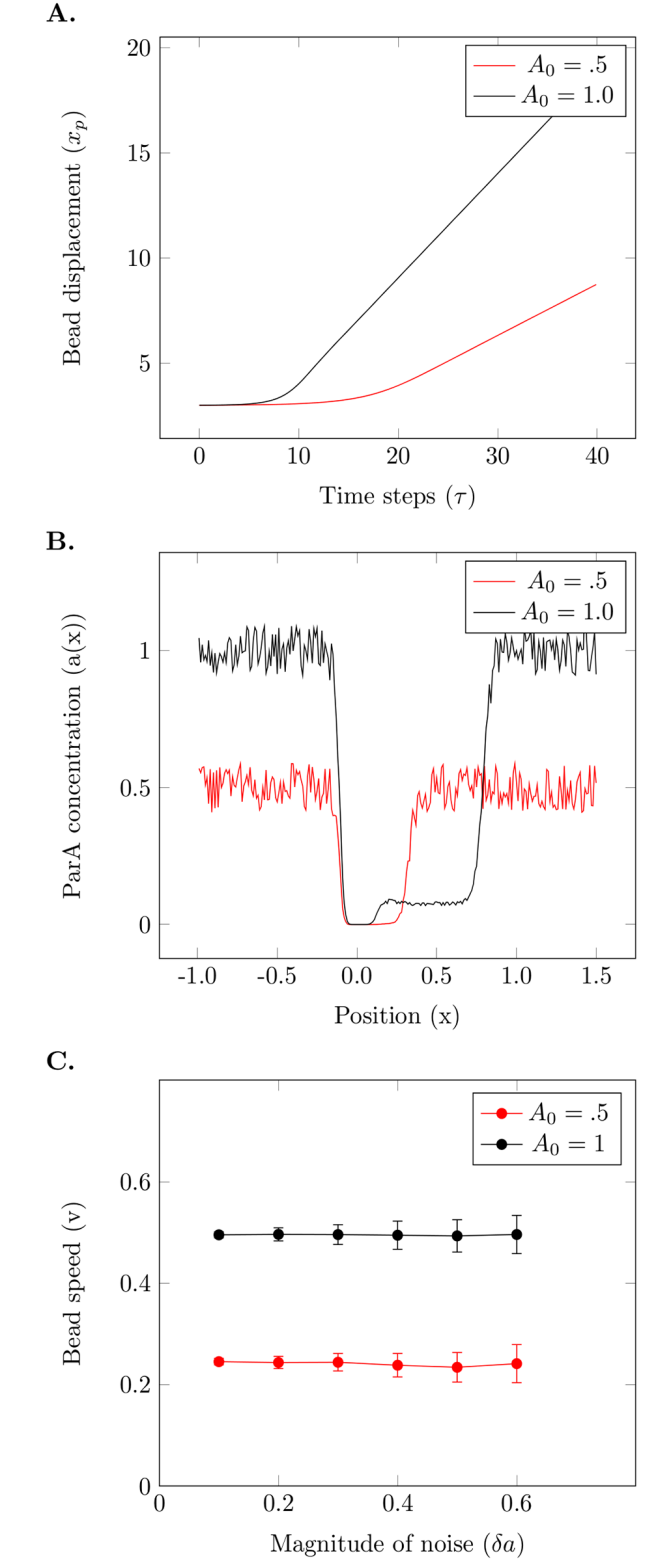
Spatial noise in the initial ParA concentration is sufficient to generate directed motion. (A) Bead displacement versus time shows an initial lag period where there is no movement. At later times, the bead attains a constant speed as evidenced by the linear increase of displacement with time. The speed is larger for the system with larger *A*_0_. (B) Simulated ParA profiles for a bead starting at *x*_*p*_ = 0 is shown after 450 time steps. The simulation with higher average initial ParA shows greater bead displacement. (C) The speed of the bead is directly proportional to *A*_0_ and does not depend on the magnitude of noise. The error bars for each point are calculated from 100 simulations. The value of *c* for these simulations is 0.5.

The only source of noise in our system is in the initial conditions that describe the ParA concentration at every point. Starting with the bead at rest, integrating the above deterministic equations show that after a short time lag the bead begins to move (Fig 2A). This lag period has also been observed in the *in vitro* experiments [21]. The cause of this movement is the non-zero net force that builds along a particular direction due to the noise in the initial bound ParA distribution which breaks the symmetry around the bead. As the bead travels it removes ParA-ATP leaving a wake behind itself and creates a wavefront of bound ParA in front (Fig 2B). Since the bead is over-damped it has no inertia from its previous step and movement along the chosen direction is sustained because the backward pulling force due to the ParA behind the bead is less than that due to the ParA in front.

**Fig 3:**
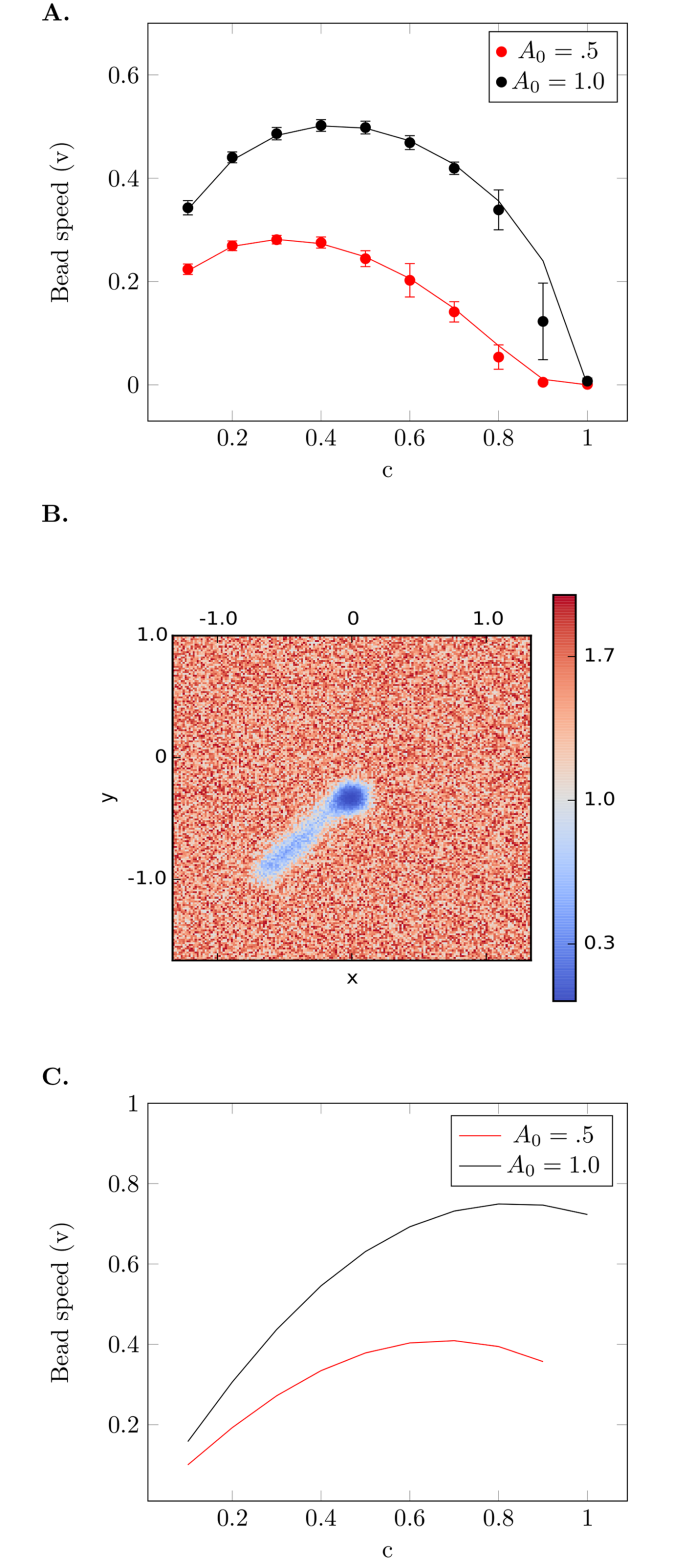
Dependence of bead speed on *c*. (A) Steady state speed of the bead as a function of *c* in 1d was calculated analytically (see S1 Text) for *A*_0_ = 0.5 (red line) and *A*_0_ = 1.0 (black line). Overlaid are the results of numerical simulations for *A*_0_ = 0.5 (red circle) and *A*_0_ = 1.0 (black circle) computed over 100 runs (error bars are the standard error of the mean). The speed peaks at a particular value of *c*, which depends on the parameter *A*_0_. (B) Heat map of the ParA concentration on a 2d substrate after 150 time steps for a bead starting at the center. There was no rebinding in this simulation, and so the wake is clearly visible. (C) Steady state speed of the bead as a function of *c* on a two dimensional surface calculated analytically (see S1 Text) for *A*_0_ = 0.5 (red line) and *A*_0_ = 1.0 (black line).

In two dimensions, a similar lag period is observed during which the bead reduces the ParA concentration (Fig 3B). After symmetry breaking the bead maintains motion along a particular direction as the attractive forces along the sides of the bead roughly cancel out whereas the forward motion is sustained due to the depleted concentration behind the moving bead.

### Dependence of bead’s speed on system parameters

For the model given by Eqs 1 and 2, following the symmetry breaking the bead attains a uniform speed as can be seen by the linear displacement of the bead with time (Fig 2A). The average speed is independent of the magnitude of the noise in the initial ParA distribution (Fig 2C), though we find that the standard deviation of the speed does increase with the noise level. We also find by increasing *A*_0_ (for example by increasing the initial mean ParA concentration), the bead’s speed increased, keeping c fixed. The constant of proportionality between speed and *A*_0_ is found to be dependent on *c*.

Given that the bead attains a uniform speed we analytically solved Eqs 1 and 2 in this limit (see S1 Text). The steady state ParA distribution for a bead moving with constant speed was found that was then used to solve for the steady state net force acting on the bead (see S1 Fig). This force balances the drag force on the bead moving at constant speed, *v*, that leads to a non-linear equation that can be solved for the speed in terms of the two free parameters, *A*_0_ and *c*.

In Fig 3A, we show that the analytical results for the predicted speed, *v*, as a function of *c*, match well with the simulated results found from integrating Eqs 1 and 2. We observe that the speed of the bead is maximized at a particular value of *c* for a fixed value of *A*_0_ and this value of *c* at which the speed is maximized changes with changing *A*_0_. The presence of a maximum speed was not unexpected, since in the limit *c* → 0, no ParA is removed and hence there is no motion and when *c* → 1, too much ParA is removed and the gradient is weakened, lessening the speed.

The two dimensional simulation shows a typical trajectory on a 2d substrate that has a noisy initial distribution of ParA (see Fig 2B). Now the bead can spontaneously move in any direction and moves roughly in a directed fashion over the surface. Similar dependences on *A*_0_ and *c* were also found. The analytical solution was extended to 2d by considering the entire speed of the bead to be along an axis (see S1 Text for details). It predicted similar features of maximum speed at a particular value of *c*, which was confirmed by simulation. Interestingly, for values of *c* less than ∼ 0.3 the bead is faster when placed in a 1d system. But for values of *c* greater than ∼ 0.3 the bead would attain higher speed on a 2d surface (Fig 3C). This can be intuitively understood as follows: when *c* < 0.3 on a 2d surface the wake of reduced ParA behind the bead is thinner than the bead’s effective radius that is attracted by the remaining ParA. This implies that there is a backward pulling force component due to unremoved ParA behind the bead. This force component decreases as *c* > 0.3 and the wake width nears the effective bead radius. This leads to lower speeds for *c* < 0.3 and higher speeds for *c* > 0.3 in 2d.

### Including ParA rebinding leads to two dynamical regimes

**Fig 4:**
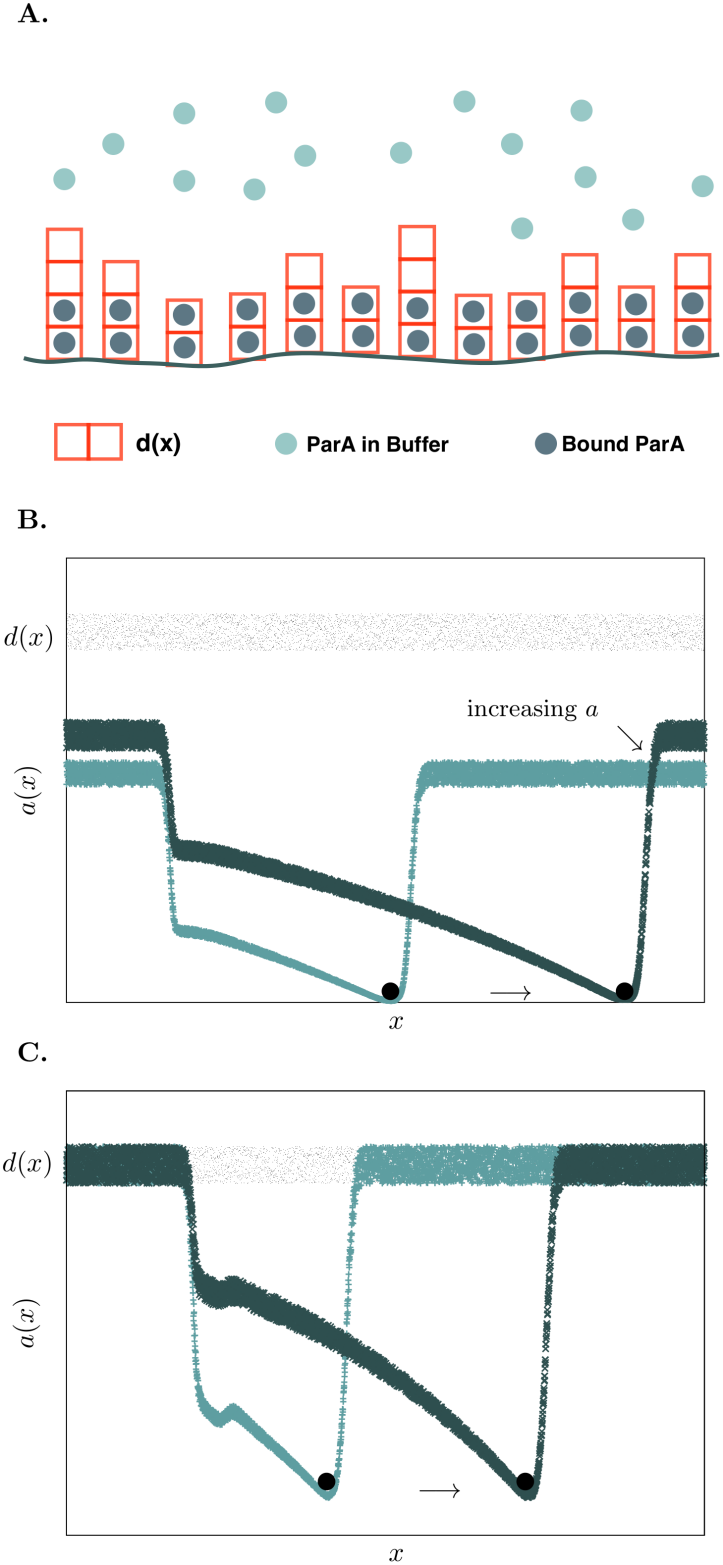
Rebinding of ParA proteins leads to two dynamical regimes. (A) Schematic for the model including rebinding. Now the DNA surface is described by a concentration of binding sites for ParA at a given position, *d*(*x*). From position to position it varies around an average concentration of sites given by *D*_0_. Free ParA in the buffer, *a*_*b*_ can now bind to locations on the surface that have unoccupied binding sites. (B) Simulated results for the time evolution of the surface bound ParA for *ϕ* = *A*_*s*_/*D*_0_ < 1 (*ϕ* = 0.5) from *τ* = 60.0 (light blue) to *τ* = 100.0 (dark blue). The amplitude of the ParA wavefront created by the bead rises with time, causing the bead’s speed to increase. (C) Simulated results of the time evolution of the surface bound ParA for *ϕ* > 1 (*ϕ* = 1.1) from *τ* = 40.0 (light blue) to *τ* = 70.0 (dark blue). Since the surface is saturated the moving ParA wavefront remains constant in height leading to the bead moving with constant speed. For these simulations only non-cooperative rebinding was considered at a rate *k*_*r*_ = 0.25 and *c* = 0.5.

Next we investigated the inclusion of ParA rebinding to a one-dimensional substrate. We assumed that at each position *x* along the surface there was a certain concentration of binding sites for ParA on the DNA substrate, *d*(*x*), whose average is *D*_0_, and fluctuates an amount *δd* (see Fig 4). We now also consider that there is ParA in the buffer, given by the quantity *a*_*b*_(*τ*). Diffusion is quick in the buffer and so ParA in the buffer does not depend on position. The amount of ParA in the system is limited by the initial amount in the buffer which is set to *a*_*b*_(0) = *A*_*s*_. For non-cooperative binding of ParA to the substrate, rebinding depends on the amount of unbound sites available at a given location, namely (*d*(*x*) − *a*(*x*, *τ*)) and the rebinding rate, *k*_*r*_. We included cooperative rebinding with a term that depends on the amount of ParA on the surface, and is governed by a cooperative rate, *k*_*c*_. Including this favors rebinding to sites that possess larger amounts of bound ParA. Including rebinding changes Eq 1 to the following equation,

$$\begin{array}{c} \frac{\partial a(x,\tau )}{\partial \tau }=-e^{-{\left(x-x_{p}\right)}^{2}/2c^{2}}a\left(x,\tau \right)+a_{b}\left(\tau \right)\left[d\left(x\right)-a\left(x,\tau \right)\right]\left[k_{r}+k_{c}a\left(x,\tau \right)\right]. \end{array}$$ (3)

While the equation for the ParA per binding site available in the buffer, *a*_*b*_(*τ*) is given by:

$$\begin{array}{c} \frac{da_{b}\left(\tau \right)}{d\tau }=\int dx\left(e^{-{\left(x-x_{p}\right)}^{2}/2c^{2}}a\left(x,\tau \right)-a_{b}\left(\tau \right)\left[d\left(x\right)-a\left(x,\tau \right)\right]\left[k_{r}+k_{c}a\left(x,\tau \right)\right]\right)/L \end{array}$$ (4)

Since the ParA released due to the bead diffuses rapidly in the buffer it adds to the free concentration *a*_*b*_ uniformly. As the ParA released by the bead is distributed over the entire system, the system size (*L*) and rate of rebinding (*k*_*r*_) are coupled in our model. Increasing the length of the system would effectively decrease the rate of rebinding as the ParA released would be diluted over a larger area. The finite size effects of our model also become evident when the bead nears a boundary of the surface. In the 1d model, on reaching an edge the bead stopped there until the ParA concentration was recovered enough in its wake and then it would begin to move back to the other end. In 2d, our model found that a bead would change directions when it encountered a boundary but would never come to a halt, unlike in 1D (see S2 Fig).

Depending on the initial amount of ParA in the buffer, *a*_*b*_(*τ* = 0) = *A*_*s*_, there are two possible regimes defined by the quantity *ϕ* = *A*_*s*_/*D*_0_: *ϕ* > 1 is the saturated regime where there is an excess of ParA and *ϕ* < 1 in which the system is undersaturated, and there is always an excess of binding sites for ParA.

**Fig 5:**
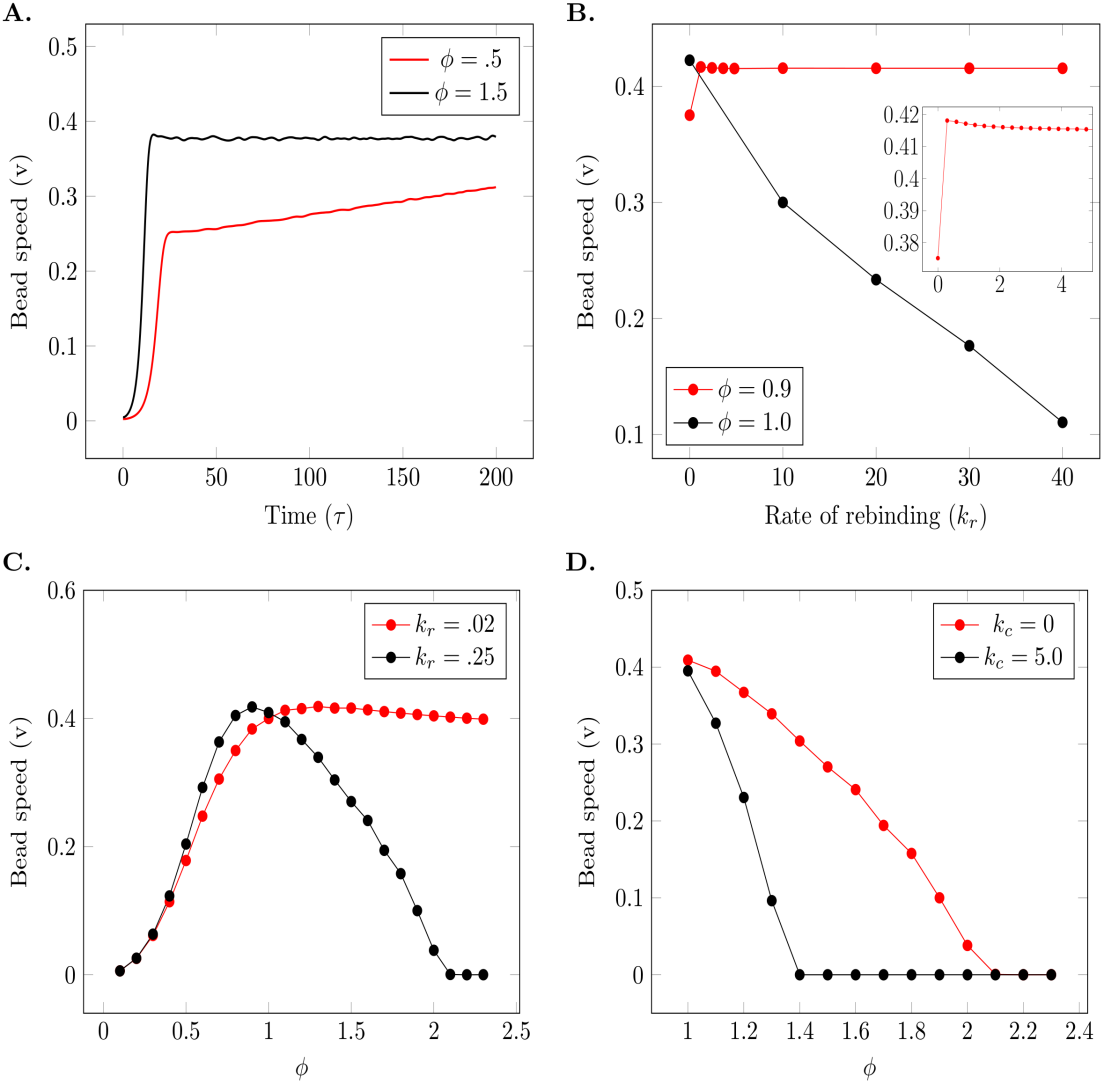
Dependence of bead speed on *ϕ* and ParA rebinding kinetics. (A) Speed of bead versus time for undersaturating (*ϕ* < 1) and saturating (*ϕ* > 1) ParA concentrations. When the system is saturated, the bead attains a constant speed. In the undersaturated conditions, the bead shows a period of persistent acceleration. For both cases, non-cooperative rebinding of ParA was used with a rate of *k*_*r*_ = 0.25 and *c* = 0.5. (B) The dependence of the final speed attained by the bead on the rate of rebinding, *k*_*r*_. When *ϕ* ⩾ 1 increasing *k*_*r*_ reduces the final speed of the bead as there is increased ParA recovery in the wake region created by the bead. For *ϕ* ≲ 1, the final speed of the bead is greater than the case without any rebinding present (*k*_*r*_ = 0) and reduces to a constant value as *k*_*r*_ is increased (inset). (C) Dependence of the bead’s speed on the non-cooperative rebinding rate and *ϕ*. In the saturating regime (*ϕ* > 1) the bead can stall if the rebinding rate is sufficiently large (black curve with *k*_*r*_ = 0.25) (D) Inclusion of cooperative rebinding (black curve with *k*_*c*_ = 5.0) can stall the bead at lower values of *ϕ* compared to non-cooperative rebinding alone (red curve with *k*_*c*_ = 0.0).

When the system was initialized with limited ParA such that *ϕ* < 1, there was still the possibility of further rebinding at every point. On introducing the bead into the system, a spontaneous gradient forms and the bead starts traveling in a particular direction as in the previous sections. As the sites in front of the bead have the capability to bind more ParA, the wavefront attracting the bead increases in magnitude and the bead gains further speed (Fig 4B). Thus in the undersaturated regime, we predict that it may be possible to observe persistent acceleration of the bead (Fig 5A).

In the regime *ϕ* > 1, there is excess ParA in the buffer and during the equilibration phase (before the ParB decorated bead is introduced), the bound ParA is nearly equal to the saturating limit *d*(*x*) while free ParA in the buffer still remains. Bead motion in this regime is similar to that described in the first section. A wavefront forms due to hydrolyzation and as all sites ahead of the bead are saturated the leading edge can not grow (Fig 4C). Indeed, we find that the bead attains a uniform speed, experiencing only an initial short burst of acceleration.

Within these two regimes a variety of bead behaviours can be observed on varying *ϕ*. In the undersaturated regime, when *ϕ* ≪ 1 the bead accelerates persistently, never reaching a saturated speed within the surface length *L*. We carried out a simple analytical calculation (see S3 Text) in this limit to determine the dependence of the bead’s speed on time which matched our simulation results (see S3 Fig). For values of *ϕ* ≲ 1 it is possible for the bead’s speed to increase and saturate to a constant value. This happens when the bound ParA wavefront in front of the bead rises to saturate all the binding sites. In the oversaturated regime, when *ϕ* ⩾ 1 there is a possibility of the buffer ParA filling the wake region completely. If this occurs rapidly enough, we expect that there should be potential to stall the bead. For a fixed value of *k*_*r*_, we observed that increasing the *ϕ* led to reduced bead speeds until a value *ϕ*_*stop*_ was reached, at which the bead did not commence motion (Fig 5C).

Besides depending on *ϕ*, the dynamics of the bead also depend on the rate of rebinding, *k*_*r*_. We find that in the undersaturated regime when *ϕ* ≲ 1 the final value to which the bead speed tends, decreases with increasing *k*_*r*_ to a saturated value (Fig 5B inset). Hence, it is not possible to stall the bead no matter how high the rate of the rebinding is. In the saturated regime however the bead can be made to stop by increasing *ϕ* and this *ϕ*_*stop*_ depends on *k*_*r*_. Some analytics that show this dependence are given in the S3 Text section of this paper and agree well with our simulated results (S3 Fig).

### Cooperative rebinding versus non-cooperative rebinding

We then considered the case of cooperative rebinding, where we assumed that rebinding depended not only on the amount of free binding sites (*d*(*x*) − *a*(*x*, *τ*)) but also on the amount of ParA bound at a given location. This cooperative rebinding was governed by a rate constant, *k*_*c*_ while a non-zero *k*_*r*_ gave the rate of non-cooperative rebinding from the buffer (Eq 3).

In the regime where *ϕ* < 1 the bead behaviour was similar for simulations with and without any cooperativity in rebinding. As the amount of ParA in the buffer was scarce, it immediately redistributed to all unsaturated sites. In the *ϕ* < 1 regime, introducing cooperative rebinding increases the acceleration of the bead as lesser ParA rebinds to the wake and more ParA rebinds to the wavefront ahead. In the *ϕ* > 1 regime the role of the rates of rebinding become more prominent as there is ParA available for rebinding, but only in the regions from where the ParA is hydrolyzed. Increasing the cooperative rebinding rate led to the depletion zone filling in faster, making it possible for the wake to recover and stall the bead at lower values of *ϕ* (Fig 5D). Hence, introducing cooperativity in ParA rebinding led to the bead stopping at lower values of *ϕ* than when there was no cooperative binding.

### ParA surface diffusion is nearly indistinguishable from rebinding

Lastly, we considered the effect of surface diffusion of ParA in the absence of rebinding to determine if there were any significant differences in bead behaviour from that of rebinding from a well mixed buffer alone. We again included a saturating limit for the amount of ParA concentration that could exist at every point *x*. This coupled the amount of available ParA in the system to the binding site distribution, *d*(*x*) as ParA could diffuse to a point only if it had the capacity to bind more ParA. We assumed that ParA surface diffusion was governed with a diffusion coefficient, *κ*. The equation describing the bead dynamics remains the same while the equation describing *a*(*x*, *τ*) in the presence of surface diffusion and no rebinding is given by (see S2 Text for details):

$$\begin{array}{c} \frac{\partial a(x,\tau )}{\partial \tau }=-e^{-{\left(x-x_{p}\right)}^{2}/2c^{2}}a\left(x,\tau \right)+\kappa \left[d\left(x\right)\frac{\partial^{2}a\left(x,\tau \right)}{\partial x^{2}}-a\left(x,\tau \right)\frac{d^{2}d\left(x\right)}{dx^{2}}\right]. \end{array}$$ (5)

As the concentration of binding sites is spatially noisy this coupling maintains the spatial noise necessary for the formation of the spontaneous gradient in ParA that initiates bead motion.

**Fig 6:**
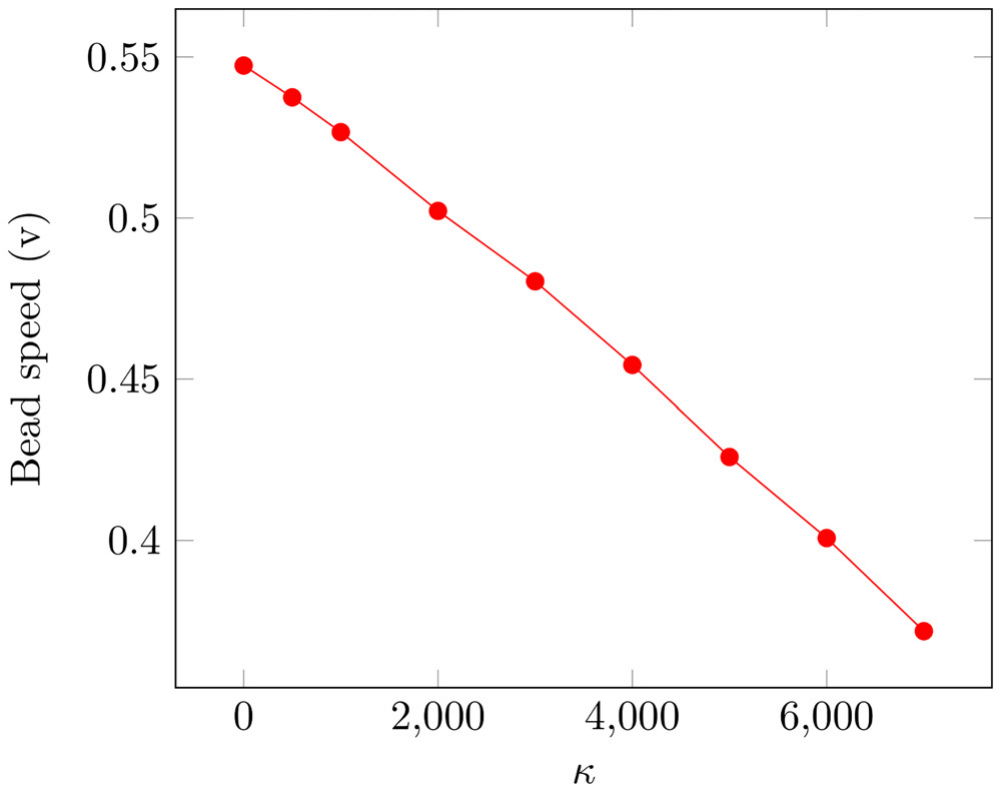
Surface diffusion of ParA reduces the speed of the bead. Dependence of the speed of the bead on the ParA protein’s surface diffusion constant, *κ*. As diffusivity increased the wake created by the bed filled in faster, reducing the total forward force. No ParA rebinding was considered. We used *ϕ* = 0.8 and *c* = 0.5.

The system was initiated in a state such that all sites on the surface have bound ParA equal to the binding distribution. When ParA protein’s surface diffusion is low, the wake can not fill in rapidly enough to stall the bead and so it attains a steady state speed. By increasing the diffusion constant, just as was the case for rebinding rates, the bead could be made to stall (Fig 6). Thus qualitatively, the behaviour is nearly identical to the situation where only rebinding occurred from the buffer.

## Discussion

In this paper we have presented a minimal model for the operation of the *in vitro* ParA-ParB system. The model involved a tug-of-war between attractive forces exerted on a ParB decorated bead by surface bound ParA in front of and behind the bead. ParB on the bead would remove ParA from the surface, tilting the balance in favor of one side, leading to directed motion. For a range of parameter values, we found that spatial noise in the initial ParA distribution was sufficient to break spatial symmetry, causing the spontaneous formation of a ParA gradient between the front and back of the bead, leading to motion.

It was found experimentally that identical beads could display different speeds when placed on the same DNA substrate. Our model provides some insight into parameters that influence a bead’s speed. The first such parameter is *A*_0_ which depends on both the initial ParA concentration as well as the amount of ParB on the bead. A simple explanation for the observed differences in speed, would be that the amount of ParB may not be the same on each bead, and hence, even though the surface bound ParA concentration that each bead sees is the same, the effective *A*_0_ would be different. Another contributing factor that could influence the amount of ParB on a bead that can interact with the surface, is bead bound ParA. Experimentally it was found that the ParA content on the beads undergoing directed motion was 25 ± 5% less than on beads which diffused freely [21]. This suggests that increased ParA on the bead lowers its ability to interact with surface bound ParA and transforms the bead into a freely diffusing particle. In our model, ParA bound to beads would affect *A*_0_ through the effective change in the amount of ParB that can interact with the surface.

The speed of a bead also depends on the parameter *c* which is the ratio of the lengthscale of the ParA removal kinetics to the lengthscale of the ParA-ParB attraction force. We speculate that a change in this ratio could be experimentally achieved by changing the size of the DNA linker that binds ParB to the micron-sized bead. The changes in bead speed due to different *c* could be characterized by looking at the shape and size of their wakes.

The model displayed a rich variety of behaviour when rebinding of ParA to the surface was considered. We showed that when the surface was saturated and there is always free ParA in the buffer that it should be possible to stall the bead. For the situation where the surface is unsaturated and free ParA can always find free sites to bind, we predict that persistent acceleration of the bead results, with the counter intuitive result that lesser total amount of ParA can actually lead to higher speeds. In order to potentially see acceleration, one would likely have to study beads on much narrower tracks so that the released ParA could have an appreciable effect on the wavefront when it rebinds. These predictions that only depend on the amount of ParA in relation to binding sites should be readily testable experimentally.

A topic of some debate about the operation of the ParA-ParB system is the role of cooperative binding for ParA and whether the formation of filaments or ParA clusters is essential. We included cooperative rebinding of ParA in our model and found that it was qualitatively indistinguishable from non-cooperative rebinding in regards to the bead dynamics. More complex dynamics, that include having multiple beads (the *in vitro* version of multiple plasmids in a cell) may be able to disentangle whether cooperative rebinding has any detectable effect. This will be a topic for further exploration. We also allowed for ParA surface diffusion and found that it too led to dynamical behaviours that would be hard to distinguish it from non-cooperative rebinding.

Although our model was developed to capture essential features of the *in vitro* ParA-ParB system, we feel that it may serve as a useful coarse grained model for studying the *in vivo* system. Future work towards this end would allow for ParB to diffuse and include discretizing the system to consider stochastic kinetic effects. Neither of these is currently in the continuum model presented here, but likely play a role *in vivo*. In summary, the model presented here makes several non-trivial predictions that should further aid the dissection of the operational mechanisms of this active transport system.

## Methods

### Dedimensionalizing deterministic model for *in vitro* ParA-ParB dynamics

To derive the dimensionless Eqs 1 and 2 we start with a 1d version of our model in real space-time coordinates *X* and *t*. The concentration of ParA at every point on the surface is given by *A*_*m*_(*X*, *t*) which can only be removed by the ParB decorated bead. The rate of removal depends on the bead’s position, *X*_*p*_ and decays with distance from the bead. We assume that the rate has a Gaussian form, centered on the bead with a characteristic range of removal given by the parameter, *σ*_*r*_. The bead has attractive forces acting on it due to its interactions with the ParA on the surface. The force between the bead and ParA on the surface decays with distance from the bead, and is directed along the surface in proportion to the *X* component of the vector connecting the bead to the surface location. Similar to the rate of removal, we consider the magnitude of the force to have a Gaussian form with a characteristic range given by *σ*_*f*_. The total force is found by integrating over the entire surface. We consider that the dynamics of the bead is in the over damped regime and so the net force, *F* is proportional to the bead’s speed, *dX*_*p*_/*dt*. Putting all these assumptions together we start with the following dimensionful equations for the ParA-ParB system:

$$\begin{array}{c} \frac{\partial A_{m}\left(X,t\right)}{\partial t}=-\gamma_{0}\;e^{-{\left(X-X_{p}\right)}^{2}/2\sigma_{r}^{2}}A_{m}\left(X,t\right) \end{array}$$ (6)

$$\begin{array}{c} \beta \frac{dX_{p}}{dt}=F=\int dX\;F_{0}\;e^{-{\left(X-X_{p}\right)}^{2}/2\sigma_{f}^{2}}\frac{X-X_{p}}{\sqrt{R^{2}+{\left(X-X_{p}\right)}^{2}}}A_{m}\left(X,t\right). \end{array}$$ (7)

Here *γ*_0_ gives the rate of ParA removal, *F*_0_ is a multiplicative constant that scales the force per unit ParA and ParB concentration exerted on the bead and *β* is the drag coefficient of the bead given by *β* = 6*πηR*. The above equations are reducible to a dimensionless version by making the following transformations:

$$\begin{array}{c} X\to Rx,\;X_{p}\to Rx_{p},\;t\to \tau /\gamma_{0} \end{array}$$ (8)

$$\begin{array}{c} A_{m}\left(X,t\right)\to a_{0}a\left(x,\tau \right) \end{array}$$ (9)

Where *x*, *x*_*p*_ and *τ* are dimensionless variables and *a*_0_ is a multiplicative constant to scale the ParA in the system. Under these transformations and introducing the ratio *c* = *σ*_*r*_/*σ*_*f*_ the dimensionless equations governing the dynamics become:

$$\begin{array}{c} \frac{\partial a(x,\tau )}{\partial \tau }=-e^{-{\left(x-x_{p}\right)}^{2}/2c^{2}}a\left(x,\tau \right) \end{array}$$ (10)

$$\begin{array}{c} v=\frac{dx_{p}}{d\tau }=A_{0}\int dx\;e^{-{\left(x-x_{p}\right)}^{2}/2}\frac{x-x_{p}}{\sqrt{1+{\left(x-x_{p}\right)}^{2}}}a\left(x,\tau \right) \end{array}$$ (11)

Here *A*_0_ = *F*_0_ *a*_0_/*βR* and *c* are the primary parameters of the system on which *v* depends. It should be noted that varying the constant *A*_0_ might imply varying the magnitude of initial ParA concentration *a*_0_ or magnitude of the force of attraction exerted by the ParA per unit ParB present on the bead, *F*_0_ or inversely varying the radius of the bead *R*. All these dependencies have been suitably combined into the single dimensionless parameter *A*_0_, which when varied reflects changes in concentration of initial ParA, since both the strength of the attractive force and radius of the bead are assumed fixed and not readily changeable. Apart from *A*_0_ the only other parameter affecting bead speed is the ratio, *c*.

We numerically integrate the above equations for a 1d system with surface length *L* = 70 and spacing *dx* = 0.02 using the Euler step method in steps of *dτ* = 0.01. For each simulation we fixed an average initial ParA concentration and obtained the ParA concentration for every site by adding uniform noise with a magnitude *δa*. The resultant profile was a spatially noisy distribution about the mean ParA concentration. The bead was placed in the center of this surface to replicate the *in vitro* experimental process such that the bead is surrounded by ParA in all directions. The forces attracting the bead from the ParA are integrated using Simpson’s rule to obtain the total vector force on the bead and the change in its position can be calculated using Eq 2. Bead speeds were obtained by doing a linear fit to the position vs. time graphs, ignoring the initial lag period.

Similar transformations can be extended to obtain the following set of equations for a 2d system:

$$\begin{array}{c} \frac{\partial a(x,y,\tau )}{\partial \tau }=-e^{-{\left(r-r_{p}\right)}^{2}/2c^{2}}a\left(x,y,\tau \right) \end{array}$$ (12)

$$\left(r^{2}=x^{2}+y^{2}\;,\;{r_{p}}^{2}=x_{p}^{2}+y_{p}^{2}\right)$$

$$\begin{array}{c} \frac{dx_{p}}{d\tau }=A_{0}\int \int dxdy\;e^{-{\left(r-r_{p}\right)}^{2}/2}\frac{x-x_{p}}{\sqrt{1+{\left(r-r_{p}\right)}^{2}}}a\left(x,y,\tau \right) \end{array}$$ (13)

$$\begin{array}{c} \frac{dy_{p}}{d\tau }=A_{0}\int \int dxdy\;e^{-{\left(r-r_{p}\right)}^{2}/2}\frac{y-y_{p}}{\sqrt{1+{\left(r-r_{p}\right)}^{2}}}a\left(x,y,\tau \right) \end{array}$$ (14)

## Supporting Information

S1 Text (PDF)

S2 Text (PDF)

S3 Text (PDF)

S1 Fig Graphical representation of analytical solution. (A) ParA profile obtained from S1(Eq. 3) for 1d is shown at *τ* = 0 for *c* = 0.5 and *v* = 1.0. A wavefront going from 0 to 1 centered at *x* = 0 is observed. This wavefront shifts right by length *vτ* in time *τ*. (B) The force exerted on the bead along *x* modulated by the Gaussian function for the ParA profile on left. The forward force peak is higher than the backward pulling force minimum, leading to a positive force when integrated along *x*. (C) 2d ParA profile obtained from Eq. 19 is shown at *τ* = 0 for *c* = 0.5 and *v* = 1.0. The bead has reached the center of the surface creating a ParA deficient wake behind itself. The entire speed of the bead is assumed to be along *x* for simplicity. (D) The force exerted on the bead along the surface from every point modulated by the Gaussian function for the ParA profile on left. Vector integration of this surface gives constant *f*_*x*_ and *f*_*y*_ = 0.
(TIF)

S2 Fig Numerical simulations for rebinding in 2d. (A) On a surface equilibrated with *ϕ* = 0.95 the bead commences motion at *x*, *y* = 0.0, *τ* = 10 and creates a ParA wake behind itself (*c* = 0.5). (B) The ParA wake fills up as released ParA rebinds to the ParA deficient regions with *k*_*r*_ = 1.75 and the effect of finite boundaries is observed as the bead reflects back into the ParA enriched zone as it is directly attracted to it.
(TIF)

S3 Fig Agreement between deterministic simulations and analytical derivations for system parameters in the rebinding model. (A) Black markers show the increase of bead speed in the low *ϕ* limit (*ϕ* = 0.2) through simulation while red line plots the analytical function *v*_0_ *L*/(*L* − *v*_0_ *τ*), for a suitably selected *v*_0_ = 0.08 (*k*_*r*_ = 1, *c* = 0.5, *L* = 20). (B) Black markers show the simulated dependence of *ϕ*_*stop*_ on *k*_*r*_ while red markers plot 1/ *k*_*r*_ *Δτ* + 1 for *Δτ* = 2.8.
(TIF)
