# Supplementary material for: Operational Principles for the Dynamics of the In Vitro ParA-ParB System
Source: PLoS Comput Biol. 2015 Dec 15;11(12):e1004651. doi: 10.1371/journal.pcbi.1004651 (PMC4699459; doi:10.1371/journal.pcbi.1004651)
Supplement: S1 Text — (PDF) [file pcbi.1004651.s001.pdf]

S1 Text

Steady state solution for the motion of the bead. In this section we present the analytical solution to our model at steady state which gives the dependence of the bead's final speed on c and A_0. From our numerical solutions to Eqs. 1 and 2 from the main text, we find that at long times the bead attains a constant speed. Consider a bead moving with constant speed v (in x space; for real space the speed $= vR$) which starts at $x_p = -\infty$ at $\tau = -\infty$. The bead position for such a bead(with $x_p = 0$ at $\tau = 0$) is given by:

$$x_p(\tau) = v\tau \tag{1}$$

On substituting this expression in Eq. 1 (Main Text) we obtain the following expression for $a(x, \tau)$ at any time τ:

$$\frac{\partial a(x,\tau)}{\partial \tau} = -e^{-(x-v\tau)^2/2c^2} a(x,\tau) \tag{2}$$

The above equation can be integrated from $\tau = -\infty$ to $\tau = 0$ using the initial condition $a(x, \tau = -\infty) = A_0$ to give $a(x, 0)$ as follows :

$$a(x,0) = A_0 \ \exp\left(\frac{c}{v}\sqrt{\frac{\pi}{2}}\left[\mathrm{erf}\left(\frac{x}{\sqrt{2}\ c}\right) - 1\right]\right) \tag{3}$$

Fig. S1 shows the above solution for the ParA profile and the resulting force exerted on the bead in 1d at $\tau = 0$ for $c = 0.5$ respectively. The figure shows the change in amplitude of the ParA concentration at $x = 0$ as a wavefront created by the bead as it clears out ParA. This expression for ParA can be used in Eq. 2 (Main Text) at time $\tau = 0.0$ for the bead Brownian dynamics as follows:

$$v = \int_{-\infty}^{\infty} dx \ e^{-x^2/2} \frac{x}{\sqrt{1+x^2}} \left[A_0 \ e^{\frac{c}{v}\sqrt{\frac{\pi}{2}}\left[\mathrm{erf}\left[\frac{x}{\sqrt{2}\ c}\right] - 1\right]}\right]. \tag{4}$$

This is a transcendental equation for v. At a fixed value for c, we use the Newton-Raphson method to solve for the value of v.

Similar to the solution in 1d, in 2d on assuming that all the bead speed is along x (ie. $v_x = v$) and $v_y = 0$ the profile for the ParA concentration is given by integrating Eq. 12 (Main Text) as:

$$a(x,y,\tau=0) = A_0 \exp\left[e^{-y^2/2c^2} \ \frac{c}{v}\sqrt{\frac{\pi}{2}}\Big(\mathrm{erf}\left(\frac{x}{\sqrt{2}c}\right) - 1\Big)\right] \tag{5}$$

Fig. S1 shows the ParA profile in two dimensions and the force profile for the bead evaluated at $\tau = 0$ for $c = 0.5$. Again as in the 1d case the expression for ParA profile can be be integrated to give the force acting on the bead at a particular time point. Since all the bead speed is along x, substituting the ParA profile in Eq. 13 (Main Text) leads us to:

$$v = \int_{-\infty}^{\infty}\int_{-\infty}^{\infty} dxdy \ e^{-x^2/2}\Big(\frac{x}{\sqrt{1+r^2}}\Big) A_0 \exp\Big[e^{-y^2/2c^2} \ \frac{c}{v}\sqrt{\frac{\pi}{2}}\Big(\mathrm{erf}\left(\frac{x}{\sqrt{2}c}\right) - 1\Big)\Big]. \tag{6}$$

Here $r^2 = x^2 + y^2$ as before.
