# Supplementary material for: Operational Principles for the Dynamics of the In Vitro ParA-ParB System
Source: PLoS Comput Biol. 2015 Dec 15;11(12):e1004651. doi: 10.1371/journal.pcbi.1004651 (PMC4699459; doi:10.1371/journal.pcbi.1004651)
Supplement: S2 Text — (PDF) [file pcbi.1004651.s002.pdf]

S2 Text

Deterministic equations for surface diffusing ParA. Here we derive the equations presented in the main text that describe the concentration of ParA when it is allowed to diffuse across the surface. We start by assuming the motion is only in one dimension and the amount of ParA that can bind to every point x is limited by the noisy function $d(x)$. The concentration of ParA is given by $a(x,\tau)$ which can diffuse to nearby sites $x \pm \delta x$. The rate of diffusion is given by κ. The mechanism for ParA removal due to the bead remains the same as in previous sections and is neglected for the time being as we determine the changes in concentration due to diffusion only. Based on these assumptions and using the same dimensionless parameters as before the equations describing the ParA concentration are as follows:

$$\begin{aligned}\frac{\partial a(x,\tau)}{\partial \tau} = &-\kappa a(x,\tau)[d(x+\delta x) - a(x+\delta x,\tau)] \\ &- \kappa a(x,\tau)[d(x-\delta x) - a(x-\delta x,\tau)] \\ &+ \kappa a(x-\delta x,\tau)[d(x) - a(x,\tau)] \\ &+ \kappa a(x+\delta x,\tau)[d(x) - a(x,\tau)]. \end{aligned} \quad (1)$$

Taylor expanding the above equation and simplifying leads to:

$$\frac{\partial a(x,\tau)}{\partial \tau} = \kappa\Big[d(x)\frac{\partial^2 a(x,\tau)}{\partial x^2} - a(x,\tau)\frac{d^2 d(x)}{dx^2}\Big]. \quad (2)$$

Combining this expression with the expression for the rate of ParA removal due to the bead gives the final equation describing ParA concentration on a surface including ParA diffusion as:

$$\frac{\partial a(x,\tau)}{\partial \tau} = \kappa\Big[d(x)\frac{\partial^2 a(x,\tau)}{\partial x^2} - a(x,\tau)\frac{d^2 d(x)}{dx^2}\Big] - e^{-(x-x_p)^2/2c^2} a(x,\tau). \quad (3)$$
