# Supplementary material for: Operational Principles for the Dynamics of the In Vitro ParA-ParB System
Source: PLoS Comput Biol. 2015 Dec 15;11(12):e1004651. doi: 10.1371/journal.pcbi.1004651 (PMC4699459; doi:10.1371/journal.pcbi.1004651)
Supplement: S3 Text — (PDF) [file pcbi.1004651.s003.pdf]

S3 Text

Analytics for the ParA rebinding mechanism for $\phi \ll 1$ and $\phi > 1$. Based on the initial amount of ParA in the buffer, A_s, the paramter $\phi = A_s/D_0$ leads to various dynamical regimes: for $\phi \ll 1$ the bead experiences persistent acceleration, for values of $\phi \lesssim 1$ the bead accelerates and attains a uniform speed, for $\phi > 1$ the bead has constant speed and for $\phi > \phi_{stop}$ the bead does not commence motion.

Here we derive the dependence of the bead's speed on the various system parameters for $\phi \ll 1$. In this regime there is effectively no ParA in the buffer, redistributing all its current concentration of ParA onto the surface, instantaneously and uniformly. We derive a simplified equation for the growth of the ParA wavefront and the resulting time dependence of the speed of the bead. From the numerical solutions of the full dynamical system (Eqs. 1-2, Main Text) we found that the speed of the bead was proportional to the ParA wavefront, $a(\tau)$, in front of the bead. Here we assume that the bead's speed is given by $v(\tau) = \alpha a(\tau)$, where α is the proportionality constant linking the speed to the ParA wavefront. As it moves, it clears all ParA from the wavefront, which then rapidly rebinds uniformly over the surface (which is a reasonable assumption if ϕ is small and there is no cooperativity in rebinding), causing the leading wavefront to grow.

Specifically, in a time step $\Delta\tau$ the amount of distance covered by the bead would be $v\Delta\tau$ which would remove $a(\tau)v\Delta\tau$ of ParA into the buffer. Because the instantaneous speed is linked to the ParA wavefront and the buffer is always in an undersaturated state ($a_b(\tau) = 0$), the instantaneous buffer volume of ParA is $\alpha a^2\Delta\tau$ immediately before redistribution. If the length of the surface is L, the amount rebinding to the wavefront in front of the bead would be $\alpha a^2\Delta\tau/L$. Hence, we have the amount that the wavefront increases, da, in the limit that $\Delta\tau$ is very small:

$$da = \frac{\alpha a^2 d\tau}{L} \tag{1}$$

Integrating both sides gives:

$$a(\tau) = \frac{a_{\tau=0}L}{L - a_{\tau=0}\alpha\tau} \tag{2}$$

Since we have previously shown that $v = \alpha a$ the above expression can be inverted to give the speed as a function of time and starting speed, $v_{\tau=0} = v_0$:

$$v(\tau) = \frac{v_0 L}{L - v_0\tau} \tag{3}$$

This monotonic increase in the speed is observed in the deterministic system for very small values of ϕ (Fig. S3A).

In the regime where $\phi > 1$ the long term steady state solution of the deterministic simulation shows that the buffer contains a constant amount of ParA. Because the system is over-saturated the ParA wake behind the bead can no longer be zero as the amount of ParA deposited instantaneously behind the bead is substantial. Furthermore in this regime there are no free binding sites ahead of the bead and so the wavefront is just a constant equal to $D_0 = 1.$ and does not increase with time. If a_{back} denotes the effective ParA behind the bead, then by the aforementioned argument the instantaneous speed of the bead would be given by:

$$v(\tau) = \alpha(D_0 - a_{back}(\tau)) \tag{4}$$

The buffer is a constant in this regime and a suitable approximation is that the amount of ParA in the buffer goes as $L(\phi - D_0)$. This gives the amount of ParA deposited behind the bead to be $k_r\Delta\tau(\phi - D_0)$ (k_r is the rate at which the ParA rebinds in time $\Delta\tau$) which leads to (dividing all ParA concentrations by D_0):

$$v(\tau) = \alpha(1 - k_r\Delta\tau(\phi - 1)) \tag{5}$$

This matches our findings from simulations in this regime, namely, the speed decreases as k_r and ϕ are increased. As ϕ_{stop} is that value of ϕ for which the bead does not commence motion ($v = 0$), the preceding equation gives us the following dependence of ϕ_{stop} on k_r:

$$\phi_{stop} = \frac{1}{k_r\Delta\tau} + 1 \tag{6}$$

The above expression is a good match to our results for ϕ_{stop} from our deterministic simulations. Fig. S3B shows the simulated values for ϕ_{stop} (black) and values from the above formula giving $\Delta\tau = 2.8$ (red).
