# Supplementary figures and images for: Operational Principles for the Dynamics of the In Vitro ParA-ParB System
Source: PLoS Comput Biol. 2015 Dec 15;11(12):e1004651. doi: 10.1371/journal.pcbi.1004651 (PMC4699459; doi:10.1371/journal.pcbi.1004651)

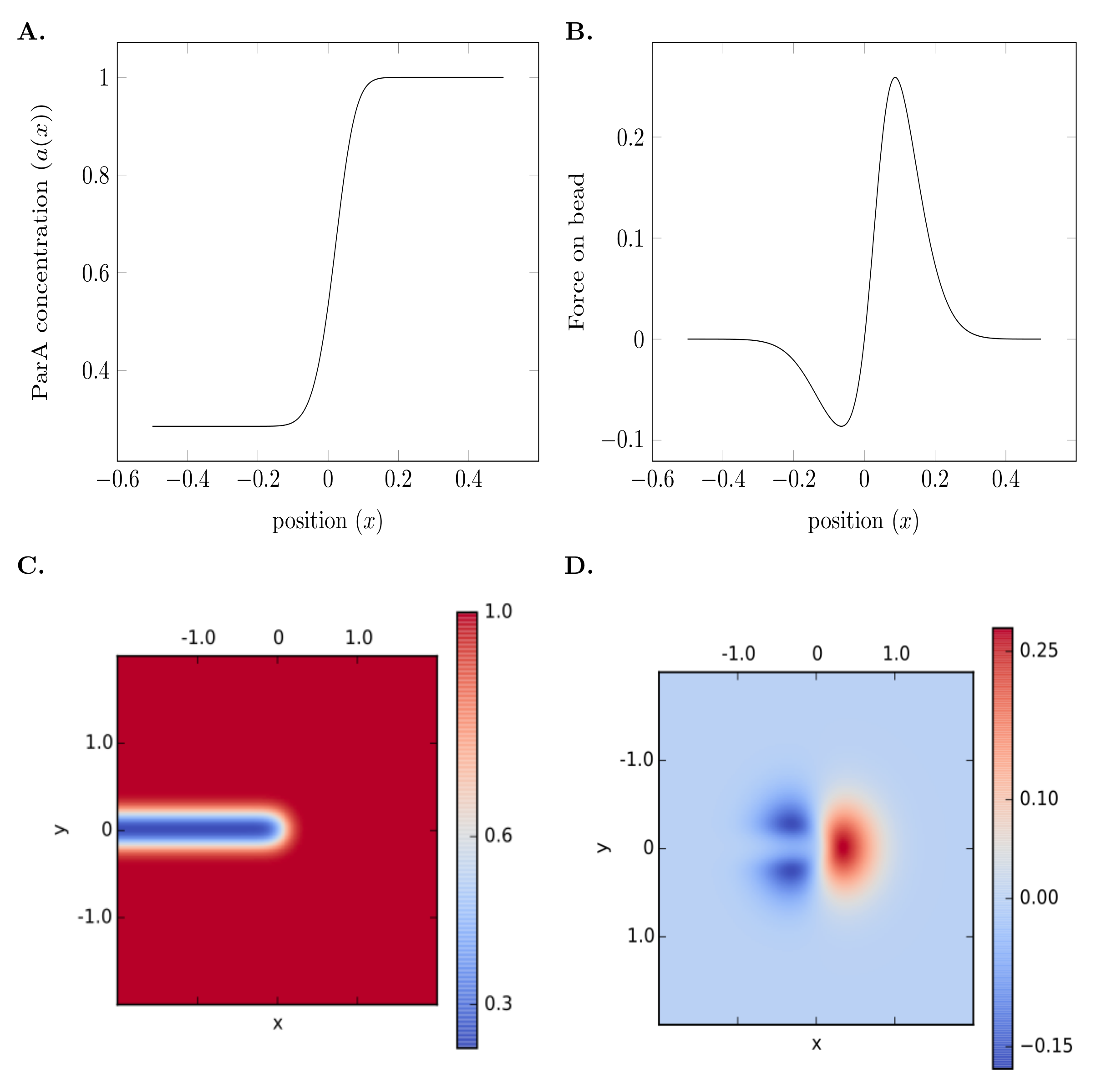

Supplement: S1 Fig — (A) ParA profile obtained from S1(Eq. 3) for 1d is shown at τ = 0 for c = 0.5 and v = 1.0. A wavefront going from 0 to 1 centered at x = 0 is observed. This wavefront shifts right by length vτ in time τ. (B) The force exerted on the bead along x modulated by the Gaussian function for the ParA profile on left. The forward force peak is higher than the backward pulling force minimum, leading to a positive force when integrated along x. (C) 2d ParA profile obtained from Eq. 19 is shown at τ = 0 for c = 0.5 and v = 1.0. The bead has reached the center of the surface creating a ParA deficient wake behind itself. The entire speed of the bead is assumed to be along x for simplicity. (D) The force exerted on the bead along the surface from every point modulated by the Gaussian function for the ParA profile on left. Vector integration of this surface gives constant f x and f y = 0. (TIF) [file pcbi.1004651.s004.tif]

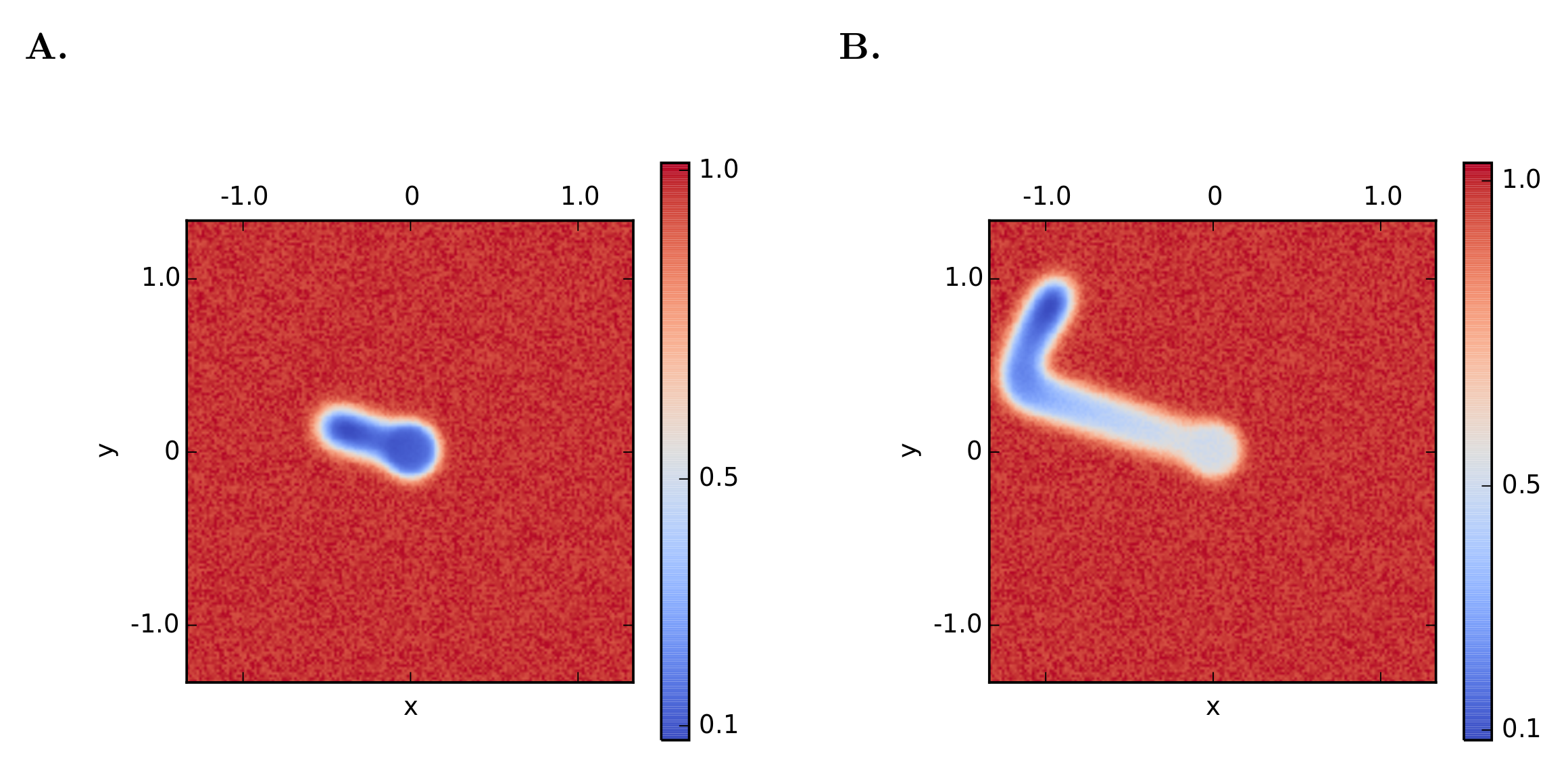

Supplement: S2 Fig — (A) On a surface equilibrated with ϕ = 0.95 the bead commences motion at x, y = 0.0, τ = 10 and creates a ParA wake behind itself (c = 0.5). (B) The ParA wake fills up as released ParA rebinds to the ParA deficient regions with k r = 1.75 and the effect of finite boundaries is observed as the bead reflects back into the ParA enriched zone as it is directly attracted to it. (TIF) [file pcbi.1004651.s005.tif]

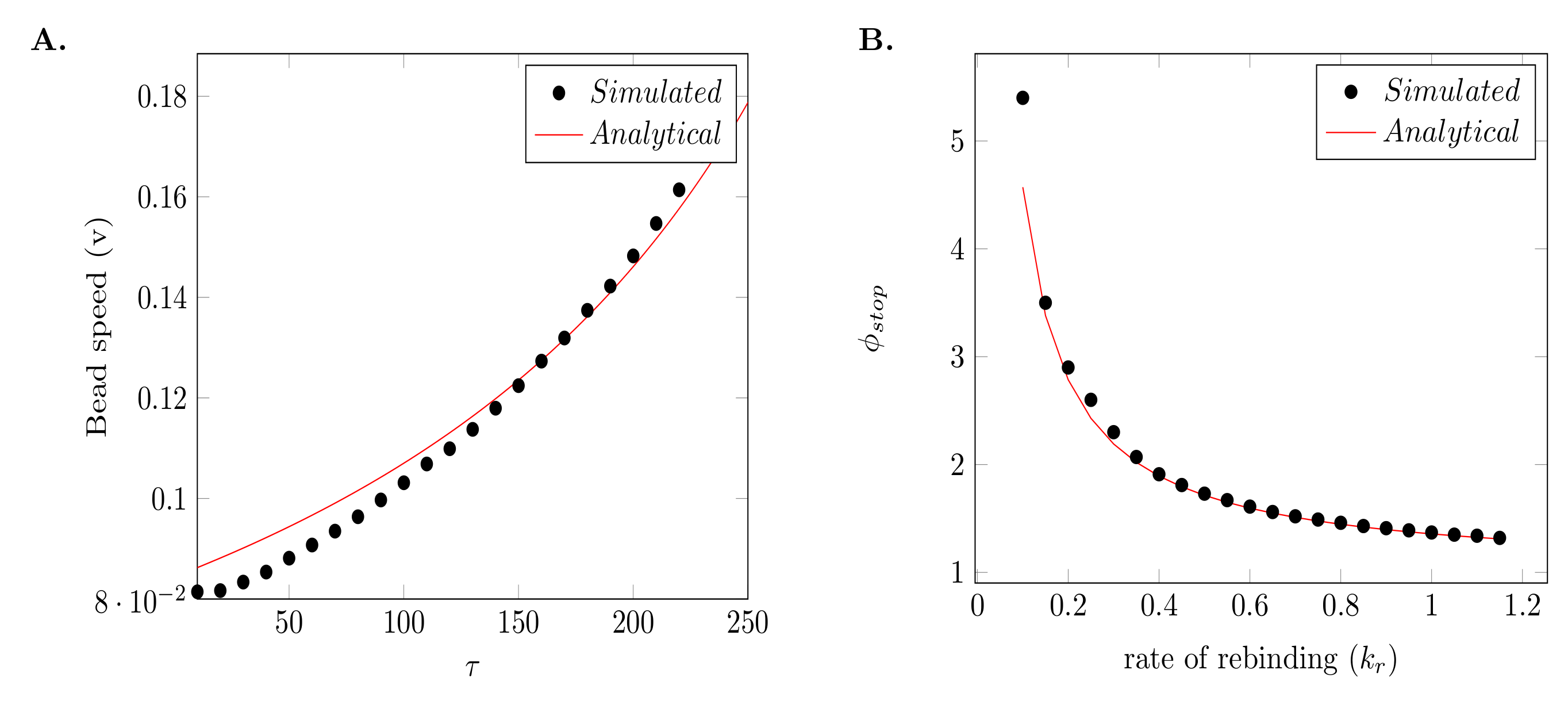

Supplement: S3 Fig — (A) Black markers show the increase of bead speed in the low ϕ limit (ϕ = 0.2) through simulation while red line plots the analytical function v 0 L/(L − v 0 τ), for a suitably selected v 0 = 0.08 (k r = 1, c = 0.5, L = 20). (B) Black markers show the simulated dependence of ϕ stop on k r while red markers plot 1/ k r Δτ + 1 for Δτ = 2.8. (TIF) [file pcbi.1004651.s006.tif]
